# Towards reproducible research in recurrent pregnancy loss immunology: Learning from cancer microenvironment deconvolution

**DOI:** 10.3389/fimmu.2023.1082087

**Published:** 2023-02-23

**Authors:** Martina Betti, Enrico Vizza, Emilio Piccione, Adalgisa Pietropolli, Benito Chiofalo, Matteo Pallocca, Valentina Bruno

**Affiliations:** ^1^ Biostatistics, Bioinformatics and Clinical Trial Center, Istituto di Ricovero e Cura a Carattere Scientifico (IRCCS) Regina Elena National Cancer Institute, Rome, Italy; ^2^ Gynecologic Oncology Unit, Department of Experimental Clinical Oncology, Istituto di Ricovero e Cura a Carattere Scientifico (IRCCS) Regina Elena National Cancer Institute, Rome, Italy; ^3^ Department of Surgical Sciences, Catholic University Our Lady of Good Counsel, Tiranë, Albania; ^4^ Department of Surgical Sciences, Section of Gynecology and Obstetrics, University of Roma Tor Vergata, Rome, Italy

**Keywords:** RPL, reproductive immunology, transcriptomics, bioinformatics, cancer microenvironment

## Abstract

The most recent international guidelines regarding recurrent pregnancy loss (RPL) exclude most of the immunological tests recommended for RPL since they do not reach an evidence-based level. Comparisons for metanalysis and systematic reviews are limited by the ambiguity in terms of RPL definition, etiological and risk factors, diagnostic work-up, and treatments applied. Therefore, cohort heterogeneity, the inadequacy of numerosity, and the quality of data confirm a not standardized research quality in the RPL field, especially for immunological background, for which potential research application remains confined in a separate single biological layer. Innovative sequencing technologies and databases have proved to play a significant role in the exploration and validation of cancer research in the context of dataset quality and bioinformatics tools. In this article, we will investigate how bioinformatics tools born for large-scale cancer immunological research could revolutionize RPL immunological research but are limited by the nature of current RPL datasets.

## Introduction

### Pathology definition

RPL is defined as two or more pregnancy losses before the 24th week of gestation, according to the most recent international guidelines of the ESHRE (European Society of Human Reproduction and Embryology) in 2017 (1), and its prevalence ranges from 1% to 5% within fertile couples (2). Known etiological factors for RPL include infections, parental chromosomal abnormalities, endocrinological and metabolic dysfunction, autoimmune diseases, antiphospholipid syndrome, major thrombophilia, and uterine anatomical abnormalities. Furthermore, several risk factors have been proposed, such as medical and family history, age, stress, lifestyle, smoking, obesity, chronic endometritis, endometriosis (3, 4), and abnormal decidualization. Nevertheless, more than 50% of RPL cases remain unexplained (uRPL) (5).

### Immune system in pregnancy

The immune system plays a key role in each pregnancy step: initiation, propagation, and termination of pregnancy (6). At a very early phase in pregnancy, the invasion of the trophoblast and the implantation process requires a pro-inflammatory environment that must switch to immune tolerance towards the semi-allogenic fetus during the later stages of pregnancy. However, the break of this tolerance is necessary at term to induce labor. The decidualization process, which is abnormal *in vitro* in women affected by RPL (7), is crucial for pregnancy outcome since it controls not only trophoblast invasion but also confers immune tolerance towards the fetus (8). In order to regulate trophoblast invasion and to promote fetal tolerance and homeostasis, the decidua holds a unique composition of immune cells with specialized properties (6). For fetal tolerance, decidual macrophages and regulatory T cells (Tregs) are of relevance; both cell types are enriched in the decidua and with an immune regulatory profile (9). During trophoblast invasion, decidual macrophages polarization shifts from a M1-skewed phenotype to a mixed M1/M2 profile, until reaching a major proportion of regulatory M2-like phenotype at the immune-tolerance establishment (10, 11). Furthermore, Tregs show an augmented suppressive profile in the decidua (12). Accordingly, aberrant activation and polarization of both cell-types may be involved in pregnancy complications including RPL.

### RPL immunology

In uRPL, an aberrant immune response at the fetal-maternal interface has been hypothesized to be responsible for its onset. Briefly, an increased pro-inflammatory response has been observed in RPL, by dysregulation of M1/M2, Th1/Th2, and Th17/Treg balance. An increasing pro-inflammatory cytokine response (e.g., high levels of TNF, IL-6, IL-17, and IFN-γ), associated with M1, Th1 and Th17 dominance, along with a diminished Th2 responses inhibiting IL-10 and G-CSF production, could confer a lack of immunosuppression due to misregulation of Treg amounts (13). In addition, a vast range of dNK functions that are involved in the regulation of vascular remodeling, fetal growth, and immune responses in normal pregnancy may exhibit altered function in uRPL (12). Furthermore, a predisposition to break auto-tolerance has been associated with RPL *via* anti-cardiolipin antibodies (ACA), anti-nuclear antibodies (ANA), anti-ds DNA and anti-thyroid-peroxidase (anti-TPO) antibody activation. Dysregulation of the maternal immune response to fetal/trophoblast antigens has been demonstrated, including through killer immunoglobulin-like receptor (KIR), human leukocyte antigen (HLA), mannose‐binding lectin (MBL) and H-Y antigen regulation (14, 15).

### Immunity in ESHRE guidelines

ESHRE guidelines exclude most of the immunological tests recommended for RPL, likely because they do not reach an evidence-based level. This is due to the lack of a broader consensus in the RPL scientific research setting, for which most of the studies cannot be compared for meta-analysis and systematic review since their data are not homogeneous in terms of definition, etiological and risk factors, diagnostic work-up, and treatments. Yet, not all the members of the consensus beyond ESHRE guidelines 2017 perfectly agreed with this document, as claimed in the document itself (1). Indeed, a review by Li J (16)., testifies how the definition of RPL remains heterogeneous also in studies posterior to 2017. Moreover, tests proposed by ESHRE (**Table 1**) are not sufficient to predict patient prognosis, stratify them into risk categories, or provide referrals for appropriate treatments and follow-up during subsequent pregnancies (5).

**Table 1:** ESHRE Guidelines on immunological biomarkers.

	ASSOCIATION	CONTRIBUTING FACTORS	TREATMENT	Prognosis
**Anti-HY immunity**	moderate	yes	no	negative impact on future live birth
**Cytokines**	yes	unclear		unknown
**Cytokines (polymorphism)**	no			
**Antinuclear Antibodies**	most studies	probably not		unclear
**NK in PB**	weak	no	no	unclear/no
**NK cell cytotoxicity in PB**	unclear		no	no
**NK in endometrium/uterus**	weak		no	unclear

### Immunity in RPL and endometrial cancer

Immune tolerance at fetal-maternal interface shares common pathways with immune escape in endometrial cancer (EC), which leads to cancer progression. Nevertheless, what is finely regulated and limited in time and space at the maternal-fetal interface in terms of immune tolerance, is conversely aberrant and irreversible in EC immune escape (17). To investigate fetal-maternal immune tolerance and its disruption in pregnancy complications, such as uRPL, could represent a model system through which immune escape molecular pathways could be unraveled in EC to better stratify patients beyond current guidelines classification, which is still not able to accurately predict prognosis and recurrences (18). Therefore, the EC immunological background could be valuable for expanding our knowledge of immune tolerance disruption in pregnancy complications.

### RPL research methodology

**Figure 1:**
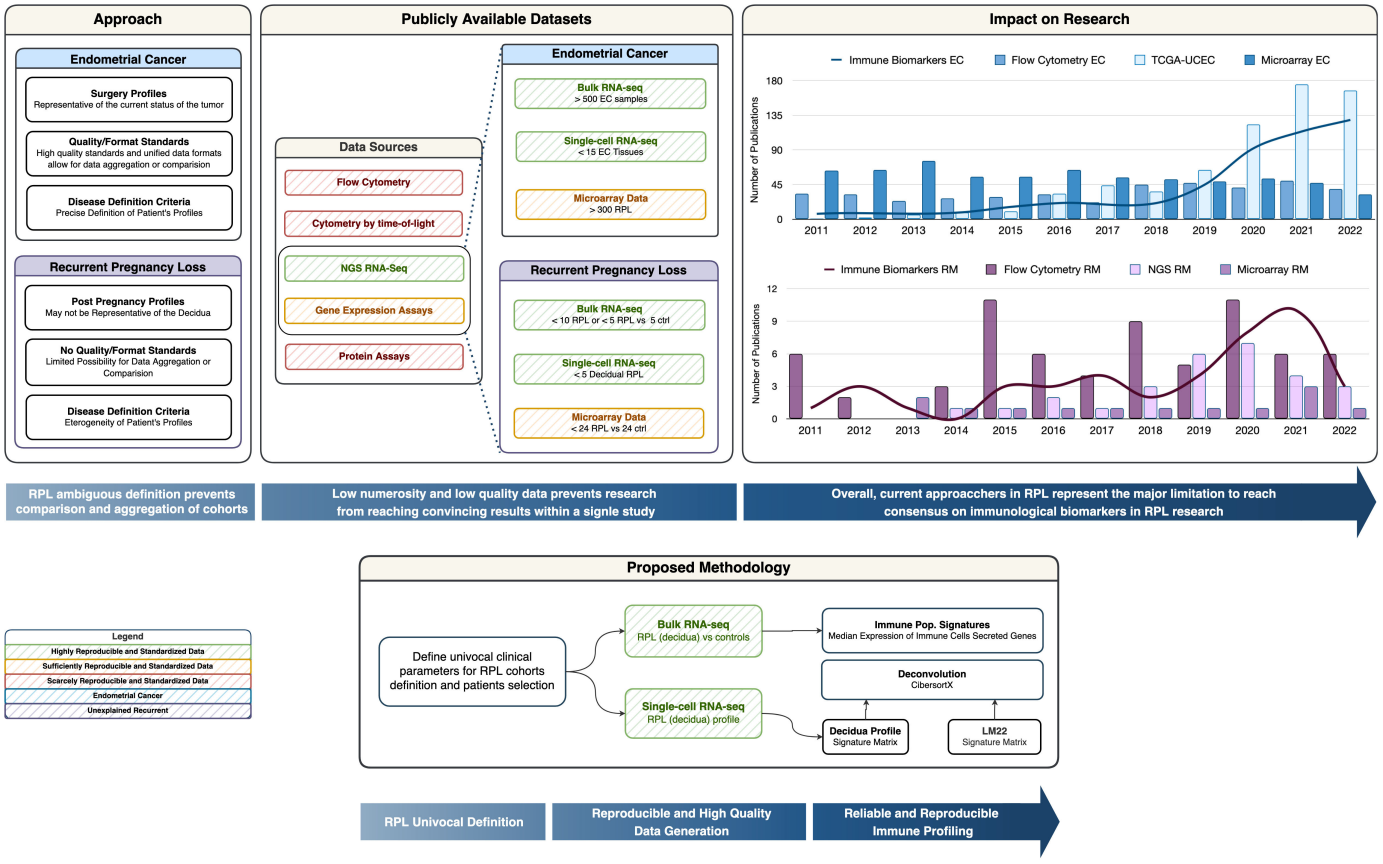
Graphical Abstract. The *Approach* box provides a summary of Recurrent Pregnancy Loss definition pitfalls compared to Endometrial Cancer Research. The *Publicly available datasets* box provides insight into the reproducible and non-reproducible technologies in the immunological field, along with a representation of the numerosity for the cohorts in RPL *vs*. EC. The *Impact on Research* box represents the trend of publication on PubMed with the keywords listed in the legend and we can observe how the number of publications on biomarkers is correlated with the biotechnological revolution. The *Proposed methodology* box represents a simplified schema of in-silico immune profiling.

From a biotechnological context, the vast majority of the available RPL experimental data is based on either signal-based-omics (e.g., microarrays) or targeted phenotypic profiles (e.g., flow cytometry). These approaches, as shown by many meta-analyses, are suffering from a lack of reproducibility or are limited by a mere phenotypic description (such as the cytokinome) without the mechanistic and complete reconstruction of the tumor-host microenvironment (19, 20) (**Figure 1**), and their level of aggregability and comparability is hindered by experiment-specific confounding and batch effects.

### Scope

In this article, we will investigate how biotechnological approaches and bioinformatics tools born for large-scale cancer immunological research could revolutionize RPL research but are limited by the nature of current RPL data.

## Methods

### RPL patient cohorts

We first sought public datasets available in the literature containing endometrial tissue RNA expression in recurrent pregnancy losses and relative controls. A review by Li J (16). et al. listed a summary of -omics RPL cohorts: although an effort is being made to reach higher numerosity and data quality, in most cases data is not publicly released. To our knowledge, only two studies have published datasets that satisfied a 10-patient-per-group constraint: RNA-seq on 10 RPL and 10 Infertile (FASTQ available at SRP052612) and Microarray Gene Expression from 24 RPL and 24 controls (series matrix GSE165004). Additionally, we searched for RPL datasets of single cell-RNA seq and found one (21) which provides the gene expression levels in the decidua of 3 RPL samples *vs*. 3 control samples. For each patient, 8 immunological populations were considered which are listed in **Table 2**. Cohort profiles and definition criteria are described in **Table 3**.

**Table 2:** List of populations considered and relative abundances in RPL.

Populations	Single Cell	Proportions (SC sig. mat.)	Proportions (LM22 sig. mat.)	Expected proportions
***Natural Killer***	**dNKe**	18%	6%	50-70%
	**dNKb**	15%		
	**dNKa**	26%		
	**dNKc**	0%		
	**dNKd**	0%		
***Macrophages***	**dM**	10%	24%	20%
***T-Cells***	**dTreg**	18%	19%	10-20%
	**dCD8T**	2%		
	**dCD4T**	12%		
***B-Cells***			10%	< 5%

**Table 3:** Cohort parameters summary.

	Microarray	RNA-Seq	Single Cell RNA-Seq
**Numerosity**	24 RPL *vs.* 24 ctrl	10 RPL	3 RPL *vs.* 3 ctrl
**Phase**	19-21 of the menstrual cycle	Mid-Luteal phase	Therapeutic termination of pregnancy
**Parity**	0	0	0
**Age**	< 35	< 40	< 37
**Comorbidities**	no	unspecified	unspecified
**Events**	3 +	3 to 8	2 +
**Consecutive events**	2 (within < 20 weeks)	unspecified	unspecified
**Normal levels/status of**	FSH, LH, E2, PRL, TSH, Leiden, prothrombin, antithrombin III, protein C and S activity, lupus anticoagulant, cardiolipin antibody, beta2-glycoprotein antibody, karyotype	unspecified	Endocrine status, chromosomal status, uterine anatomy, renal status

### Bioinformatic analysis

The proportion of immune cells can be faithfully inferred from bulk RNA-seq profiles through the employment of deconvolution algorithms. CIBERSORTx is a support vector regression-based approach for characterizing cell subsets proportions which relies either on well-known immune cell expression profiles (LM22 signature matrix) or on a custom signature matrix built on single-cell data. For each sample, immune cell sub-population proportions have been estimated with the default setup (perm: 500, rmbatchBmode: TRUE, Quantile Normalized: TRUE).

Microarray data was already quantile normalized. Single-cell data normalization is carried out by CIBERSORTx along with the generation of the signature matrix. CIBERSORTx and Seurat packages were used respectively for deconvolution and single-cell data preprocessing. Comparisons between RPL and controls were computed *via* t-test. Immune cell signatures have been computed through a weighted geometric mean.

## Results

### Differential deconvolution on endometrial cell population expression signatures

**Figure 2:**
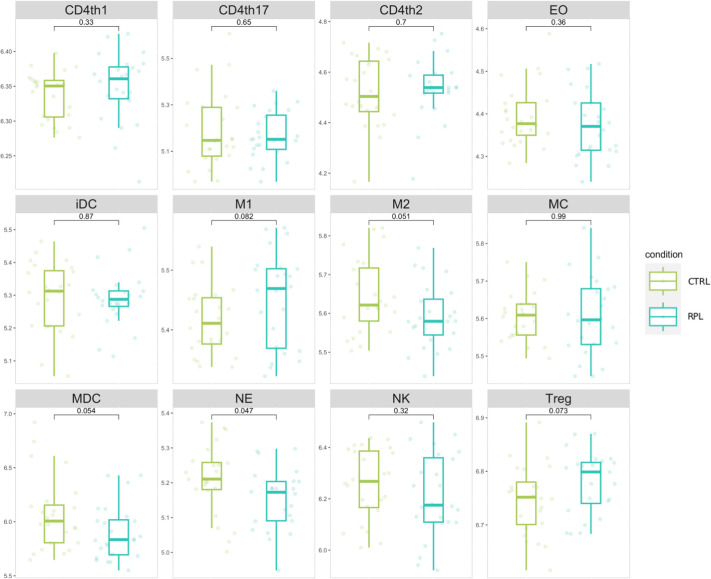
Immune cell populations signature distribution comparison. Values represent the gene weighted geometric mean of microarray expression levels, transformed to a log2 scale. The indicated p-values refer to the t-test comparison between RPL and control group.

We implemented an immune-cell activity analysis based on the expression level of genes secreted by immunological cellular subtypes according to the most recent data available from Vallvé-Juanico et. Al (22). We generated an expression signature for each immunological cellular subtype, by considering the geometric mean of expression level genes secreted by immunological cells. According to cell population expression signatures, neutrophils-specific gene expression was significantly higher in the RPL population compared to controls (**Figure 2**). Although the role of neutrophils in RPL is currently being evaluated (23), the limited abundance of these cells in the endometrium led us to interpret these findings as not representative of the immunological background in RPL. In addition, NK cell gene expression values appear to be significantly lower in comparison to other cell populations.

### CIBERSORTx deconvolution on microarray data

**Figure 3:**
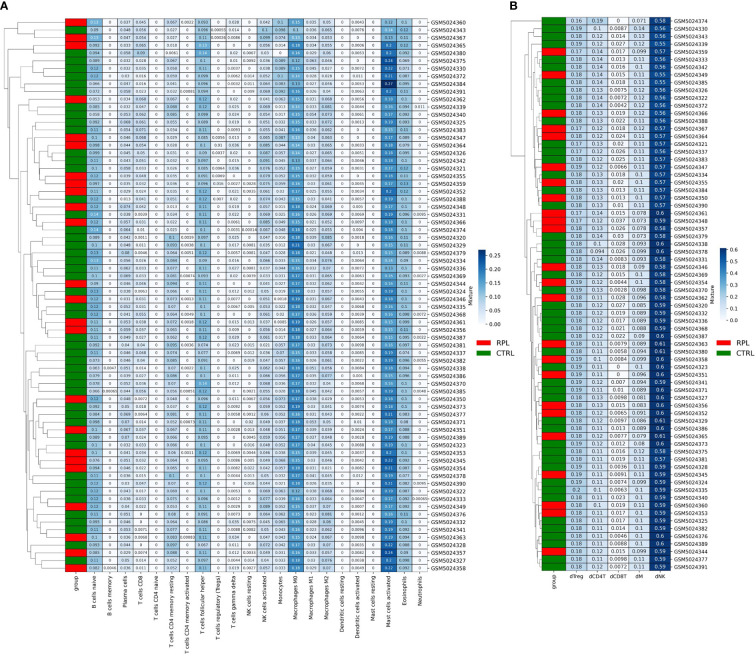
**(A)** CibersortX LM22-based Deconvolution of Microarray Expression Data. Samples do not cluster according to the RPL condition. **(B)** CibersortX cecidual immune cell-based deconvolution of microarray expression data.

We then sought more finely tuned methodologies to estimate immune cell proportions. We performed microenvironment deconvolution through the CIBERSORTx (24) software based on the LM22 signature matrix (median RMSE: 0.73) and obtained the results described in **Table 2**. A significantly higher proportion of T follicular helper cells (10.7% *vs.* 10.1% p-value: 0.002), and significantly lower proportion of M2 macrophages (5.8% *vs*. 6.0%, p-value: 0.02) were observed. These results are in line with previous reports on aberrant immune responses in RPL (25, 26). However, no evident clustering is obtained to stratify the two populations (**Figure 3A**), which is also coherent with the results obtained in other studies based on the same microarray dataset (27). We further reasoned that the limitations in describing the immune profile alteration of RPL are not related to the choice of the deconvolution method: indeed, another tool, *ImmuCellAI (* 28), has revealed similar results in other studies (27).

Since the LM22 signature matrix has been validated only on TCGA solid tumor datasets we also attempted to design an RPL-specific signature matrix based on 3 decidual single-cell profiles. For this purpose, we extracted decidual cell expression levels and calculated the mean for each immune cellular subtype for each patient. Out of the 8 original populations, only 5 were sufficiently profiled (NK cells, Tregs and other CD4^+^ T-cells, CD8^+^ T-cells, macrophages). Then, we structured the resulting data frame according to CIBERSORTx input constraints and completed the procedure as indicated in the CIBERSORTx manual. The new RPL signature matrix was used to perform the deconvolution (median RMSE: 0.91). Once again, no clustering and/or statistically significant differences in immunological populations proportions were found (**Figure 3B**), however, we observed that the immune profile described with the second approach produced more consistent results with the immune expected cell proportions (**Table 2**). Although such results could be biased by the presence of fewer immune cell populations, we believe that the peculiar secretion profile of decidual immune cells should be taken into consideration when performing deconvolution.

## Discussion

Traditional experimental assays are time-consuming, expensive, and laborious in the heterogeneity of cancer characterization. Thus, more efficient approaches have been introduced in cancer research to improve the traditional research settings to reach a precision/personalized medicine level. The availability of high-dimensional data has significantly improved cancer research and led to the employment of advanced modeling techniques, such as machine learning and deep learning. Such methodological improvements driven by cancer research have strong potential for discovery of new and complex unknown features (29), but will require meticulous standardization of quality and quantity, as for TCGA (The Cancer Genome Atlas) datasets.

Publicly available data of RPL cohorts showed quite a few limitations for data accessibility and reanalysis, for either format incompatibility with standard bioinformatics tools or quantitative/qualitative limitations of the cohorts. While the Lucas et al. NGS (30) cohort provides high quality data, no healthy control population is provided. Likewise, the microarray dataset cannot be considered satisfactory as this technology is often irreproducible (19, 20), especially due to lower abundance transcripts that have a strong overlap with genes related to the microenvironment. Further, non-solid tumor datasets may not be entirely compatible with the CIBERSORTx signature matrix (LM22) (31). As an RPL-specific signature matrix would be required for this scope, the numerosity of the RPL single-cell RNA-seq cohort was likely too scarce to represent the immune cell landscape of all patients, not to mention that the immune populations considered consist of only 5 immune cell subtypes (NK, Treg, CD4T, CD8T, M), which are rather insufficient to fully characterize the RPL immune profile.

Along with data availability limitations, one major limitation in RPL research is that separate cohorts cannot be compared nor aggregated due to the prominent level of heterogeneity in RPL definition criteria, diagnostic work-up to perform, etiological factors to investigate and their own definition, as described in Li J (16). et al. Nevertheless, it should be considered that RPL incidence is quite high in couples and has a significant impact on health systems since it affects not only the natality and psychological aspects of these women, but also their future health, since RPL women show a higher risk for obstetrical complications, cardiovascular diseases, and venous thromboembolic events later in life (32–34).

Our attempt reinforces the limitations already expressed by ESHRE 2017 guidelines on the impossibility to reach an immunological evidenced-based level, which is imputable both to the inadequacy of the *single gene* approach and the scarcity in terms of numerosity and quality of RPL studies. Research, development, and the definition of standardized procedures should be considered to produce adequate RPL databases, so to enable the application of new methodological approaches. For instance, extensive transcriptomics studies on the decidua will enable to deconvolute and pinpoint the specific cell populations that are dysregulated in RPL and compare their behavior to the best-known mechanisms of the EC immunological microenvironment.

## Data availability statement

Publicly available datasets were analyzed in this study. This data can be found here: GEO Series GSE165004 https://www.ncbi.nlm.nih.gov/geo/query/acc.cgi?acc=GSE165004 SRA SRP052612 https://www.ncbi.nlm.nih.gov/sra?term=SRP052612 https://ngdc.cncb.ac.cn/gsa-human/browse/HRA000237.

## Author contributions

VB and MB contributed to conception and design of the study. VB, MB, and MP explored the available datasets. EP, AP, and EV contributed to the clinical interpretation of the results. MB and MP performed the bioinformatics and statistical analysis. VB wrote the first draft of the manuscript. MB, and MP wrote sections of the manuscript. VB, MP, and MB reviewed the manuscript. All authors contributed to the article and approved the submitted version.
